# Biallelic *P4HTM* variants associated with HIDEA syndrome and mitochondrial respiratory chain complex I deficiency

**DOI:** 10.1038/s41431-021-00932-8

**Published:** 2021-07-20

**Authors:** Eleanor Hay, Louise C. Wilson, Bethan Hoskins, Martin Samuels, Pinki Munot, Shamima Rahman

**Affiliations:** 1 Department of Clinical Genetics, Great Ormond Street Hospital, London, UK; 2 North Thames Regional Genetic laboratory, Great Ormond Street Hospital, London, UK; 3 Department of Respiratory Medicine, Great Ormond Street Hospital, London, UK; 4 Department of Neurosciences, Dubowitz Neuromuscular Centre, Great Ormond Street Hospital, London, UK; 5 UCL Great Ormond Street Institute of Child Health, UCL, 30 Guilford Street, London, WC1N 1EH, UK

**Keywords:** Metabolic disorders, Neuromuscular disease, Sleep disorders, Disease genetics

## Abstract

We report a patient with profound congenital hypotonia, central hypoventilation, poor visual behaviour with retinal hypopigmentation, and significantly decreased mitochondrial respiratory chain complex I activity in muscle, who died at 7 months of age having made minimal developmental progress. Biallelic predicted truncating *P4HTM* variants were identified following trio whole-genome sequencing, consistent with a diagnosis of hypotonia, hypoventilation, intellectual disability, dysautonomia, epilepsy and eye abnormalities (HIDEA) syndrome. Very few patients with HIDEA syndrome have been reported previously and mitochondrial abnormalities were observed in three of four previous cases who had a muscle biopsy, suggesting the possibility that HIDEA syndrome represents a primary mitochondrial disorder. *P4HTM* encodes a transmembrane prolyl 4-hydroxylase with putative targets including hypoxia inducible factors, RNA polymerase II and activating transcription factor 4, which has been implicated in the integrated stress response observed in cell and animal models of mitochondrial disease, and may explain the mitochondrial dysfunction observed in HIDEA syndrome.

## Introduction

The differential diagnosis for neonatal hypotonia is wide as it may result from abnormality at any level of the nervous system, and the underlying basis includes both environmental and genetic causes [1, 2]. Very few causes are clinically recognisable and achieving a diagnosis requires a systematic, usually multidisciplinary, approach. Access to panel-based genetic testing and next-generation sequencing of both mitochondrial and nuclear genomes has greatly increased the potential to achieve a molecular genetic diagnosis. However, owing to the number of candidate sequence variations often yielded when multiple genes are sequenced, rigorous phenotyping is still important for the interpretation of genetic variants [3, 4].

We describe a patient presenting with a distinctive combination of persistent congenital hypotonia, central hypoventilation, abnormal visual behaviour, and minimal developmental progress. Through trio whole-genome sequencing (WGS), he was found to have novel compound heterozygous predicted truncating variants in *P4HTM (*Prolyl 4-Hydroxylase, Transmembrane). Biallelic loss of function variants in *P4HTM* have recently been reported in association with the syndrome of hypotonia, hypoventilation, intellectual disability, dysautonomia, epilepsy and eye abnormalities (HIDEA, OMIM # 618493 [5]. The findings in the patient we report support hypopigmented fundi and mitochondrial respiratory chain deficiencies in skeletal muscle as recurrent features of this syndrome that are not encapsulated in the acronym HIDEA.

## Materials and methods

Informed consent for WGS was obtained in accordance with approval from the HRA Committee East of England–Cambridge South (REC ref. 14/EE/1112). Written, informed consent was taken for publication from the patient’s legal guardians. Detailed phenotyping was undertaken by the authors of this study.

Trio (each parent and child) WGS was performed through the Genomics England 100,000 Genomes Project as previously described [6]. From the raw dataset, rare variants (de novo or biallelic) predicted to have a protein altering affect were reviewed [7]. Alamut-v.2.6 (https://www.interactive-biosoftware.com/alamut-visual/) was utilised in variant interpretation. Variants were confirmed by Sanger sequencing. Variants and phenotypic data were submitted to the Decipher database, available at https://www.deciphergenomics.org, accession number 429076.

## Results

### Clinical phenotype

The patient was the third child born to healthy unrelated Caucasian parents. The pregnancy was unremarkable with normal antenatal ultrasound scans, liquor volume, and fetal movements. Normal delivery at 38 weeks’ gestation followed induction of labour for fetal bradycardia. Birth weight was 3.97 kg and head circumference 36.5 cm (both 75th to 91st centile). He did not require any resuscitation but was noted to have a right-sided Erbs palsy, left-sided positional talipes and torticollis, which all resolved with physiotherapy. He had small medial epicanthic folds, asymmetric ears with a left extended crus helix and a small capillary haemangioma of the right thigh but otherwise his appearance was normal. He was floppy and struggled to breastfeed from birth, necessitating intermittent nasogastric feeds from 24 h of age. Although subsequently established on bottle-feeding, he fed extremely slowly and by 6 weeks his weight had dropped to the 0.4th centile. He slept for long periods, had poor tone and head control and a weak cry. His developmental milestones were globally delayed. At 10 weeks he was hospitalised for investigation of faltering growth. He was found to be in type 2 respiratory failure and was intubated and transferred to the paediatric intensive care unit where he received invasive ventilation and inotropes for 4 days. A sleep study revealed central hypoventilation with low baseline oxygen saturation (mean 93%) during quiet sleep (normal 95–100%), bradypnoea with a mean respiratory rate 16/min (normal for 6–9 weeks 27–49 breaths/min), a mixture of central and obstructive events during sleep (total Apnoea-Hypopnoea Index 3.3 events/h) and hypercapnia (>6.7 kPa/50 mmHg) during 20% of quiet sleep (Supplementary Fig. 1). He was therefore commenced on nocturnal BiPAP (Bilevel positive airway pressure ventilation). He had severe central hypotonia and head lag, right-sided ptosis, poor suck, and antigravity movements only. Knee jerks were brisk with extensor plantar responses and ankle clonus bilaterally. He passed his newborn hearing screen. He was noted to have roving eye movements, and ophthalmological assessment revealed structurally normal eyes with moderate hypermetropia, pale optic nerves and blonde fundi. Flash visual evoked potentials (VEPs) were small and suggestive of bilateral generalised visual pathway dysfunction. There was no evidence of facial weakness or tongue fasciculation. Echocardiogram, ECG, diaphragmatic ultrasound, and MRI brain were all unremarkable. The patient’s clinical features are summarised in Table 1.

**Table 1:** Phenotypic features of individuals reported with biallelic variants in *P4HTM*.

Report		Current	Rahikkala et al. [5] and Kaasinen et al. [8]											Maddirevula et al. [9]									Total (%)
Family			F1	F2		F3			F4	F5				F1			F2		F3		F4		
Predicted P4HTM protein consequence [Nationality]		p.(Gln190Leufs*9) /p.(Trp220*) [UK]	c.1073G>A (hom) predicted in-frame del exon 6 [Finnish]		p.(Gln96Profs*29) / p.(His161Pro) [North American]	p.(Gln532*) (hom) [Turkish]			p.(Val317Phefs*30) [Syrian]	c.1073G>A (hom) predicted in-frame del exon 6 [Finnish]				p.(Arg296_Arg358 delinsSer) (hom)—in-frame del exon 6 [Saudi]			p.(Arg471Ser) (hom) [Qatar]		p.(Val317Phefs*30) (hom) [Syrian]		p.(Asn274Glufs*11) (hom) [Saudi]		
			P1	P2	P3	P4	P5	P6	P7	P8	P9	P10	P11	P12	P13	P14	P15	P16	P17	P18	P19	P20	*N* = 21
Age (Age deceased)		(7 months)	(5 years)	18 years	13 years	(7 months)	(8 years)	3 years	(7 months)	31 years	29 years	18 years	21 years	55 years	(61 years)	(8 years)	7 years	?11 years	4 years	2 years	3 years	1 year	
Sex		M	F	M	M	M	M	M	M	M	F	M	M	F	F	F	F	F	M	M	F	M	M:F 13:8
Hypotonia		++	+	+	+	++	++	++	+	+	+	+	+	+	+	+	+	+	+	+	+	+	21/21 (100)
Ambulant		(NA)	+	+	+	(NA)	−	−	(NA)	+	−	−	+	+	−	−^b^	+^b^	+^b^	+^b^	−^b^	−^b^	−^b^	9/18 (50)^b^
Language		(NA)	SS	SS	NV	(NA)	NV	NV	(NA)	SS	NV	NV	NV	NV	NV	P/A	−	−	P/A	P/A	P/A	P/A	NV 8/18 (44)
ID or global delay		GD	ID	ID	ID	GD	ID	ID	ID	ID	ID	ID	ID	ID	ID	GD	ID	ID	ID	GD	GD	GD	21/21 (100)
Seizures		−	+	−	+	+	+	+	+	−	+	+	−	+	+	−	−	−	−	−	−	−	10/21 (48)
OSA		−	+	+	+	(NR)	−	−	−	−	−	+	+	NR	NR	−	−	−	−	+	−	−	6/18 (33)
Hypoventilation		+(C)	−	+(C)	+(C)	+	+(C)	+(C)	+	−	−	−	+(C)	NR	NR	−	−	−	−	+	+	+	11/19 (58)
Recurrent pneumonia		+	+	+	+	(+)	+	+	−	−	−	−	+	+	NR	−	+	−	−	+	+	−	12/20 (60)
Sleep behaviour abn		−	−	−	+	−	−	−	+	+	−	−	+	−	−	NR	NR	NR	NR	NR	NR	NR	4/14 (29)
Eye	Refractive error/strabismus	+	+	+	+	(NR)	+	+	−	+	+	+	+	+	+	+	NR	NR	NR	NR	NR	NR	13/14 (93)
	Abnormal eye movements	+	−	−	+	+	+	−	+	+	−	−	+	−	−	+	+	−	−	+	+	+	12/21 (57)
	Pale fundi	+	−	+^a^	NR	NR	+	−	NR	+	+	+	+	−	−	NR	−	−	NR	−	−	−	7/16 (44)
	Pale optic nerves	+	+	−	−	NR	−	−	NR	−	−	−	−	−	−	NR	−	−	NR	−	−	−	2/17 (12)
Thermoregulation abn		−	−	−	+	−	+	+	−	−	+	−	−	−	−	−	NR	NR	NR	NR	NR	NR	4/15 (27)
Abn muscle/RCE		+	+	+	NR	NR	+	NR	NR	NR	−	NR	NR	NR	+	NR	NR	NR	NR	NR	NR	NR	5/6 (83)
Other findings			Mild fatty liver degeneration, hyperphagia	Precocious adrenarche	Cortical blindness						Normal muscle histology. RCE-NR	Left ocular dermoid		Keratoconus	Mild fatty liver degeneration, Atrophic cerebellum		Small PDA			Hypothyroid, spinal dural AV fistula	Cortical blindness		

*Abn* abnormality, (*C*) confirmed central hypoventilation, *F* female, *GD* global developmental delay, *ID* intellectual disability, *M* male, *NA* not applicable, *NR* not reported, *NV* non-verbal, *OSA* obstructive sleep apnoea, *P/A* poor or absent speech, *Pt* patient, *RCE* respiratory chain enzymes, *SS* simple sentences.

^a^temporal disc pallor.

^b^using data from case descriptions due to discrepancies in Table 1 from Maddirevula et al. [9].

A trial of pyridostigmine and salbutamol had minimal effect. By 5.5 months of age, he was smiling responsively but had made no motor progress. No seizure activity was noted. His roving eye movements and profound hypotonia remained unchanged.

At 6 months he was readmitted in respiratory failure secondary to a viral lower respiratory tract infection. He decompensated rapidly and was admitted to intensive care where, despite a brief period tolerating BiPaP, he subsequently failed repeated attempts at extubation. Care was redirected to a palliative approach and he died aged 7 months.

### Neurometabolic investigations

Extensive neurometabolic investigations in blood (acid maltase, amino acids, ammonia, biotinidase, blood spot carnitines, caeruloplasmin, copper, creatine kinase, full blood count, glucose, lactate, liver function tests, sterols, transferrin electrophoresis, urea and electrolytes, creatinine, bone profile, vacuolated lymphocytes, very long chain fatty acids), urine (glycosaminoglycans, organic acids) and cerebrospinal fluid (amino acids, glucose, lactate, lactate/pyruvate ratio, 5-methyltetrahydrofolate, neurotransmitters, pterins, pyridoxal phosphate), and maternal acetylcholine receptor antibody testing gave results within our laboratory normal reference ranges (values available upon request).

Initial electromyography and nerve conduction studies were interpreted as suggestive, although not diagnostic, of a myasthenic disorder with the degree of jitter abnormality outside the limits expected for immaturity. A muscle biopsy performed at 6 months of age revealed mild myopathic changes including fibre size variation and a few fetal myosin positive fibres reflecting immaturity. Of note, there were no ragged red fibres or fibres staining negatively for cytochrome *c* oxidase (complex IV, COX). Spectrophotometric analysis of mitochondrial respiratory chain function identified significantly reduced complex I activity (nicotinamide adenine dinucleotide [NADH]: ubiquinone oxidoreductase) to less than 30% of the mean control at 0.054 (reference range 0.104–0.268), normal complex II and III activity at 0.078 (0.040–0.204) and marginally reduced complex IV activity at 0.012 (0.014–0.034).

### Genomic investigations

Genetic tests for common causes of neonatal hypotonia (spinal muscular atrophy, Prader–Willi syndrome, myotonic dystrophy), congenital myasthenic syndrome and central hypoventilation syndrome (*PHOX2B* polyalanine expansion mutations) were normal. There was no evidence of any detectable structural mitochondrial DNA (mtDNA) rearrangements, or mtDNA depletion, and sequencing of mtDNA and a large panel of nuclear genes associated with primary mitochondrial disease did not reveal any loss of function variants.

**Fig. 1:**
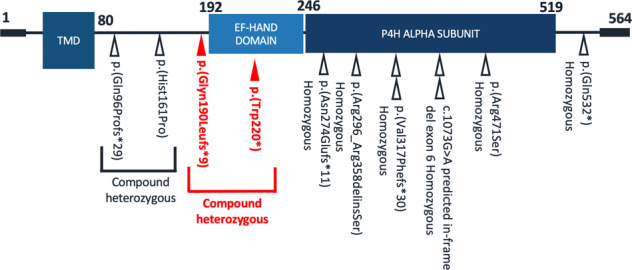
Simplified gene diagram representing the canonical transcript of P4HTM, Refseq NM_177938.2. The proband’s compound heterozygous variants are shown in bold with filled triangles (red). Previously reported variants are shown with unfilled triangles. All variants were rare or absent in population databases and predicted to result in loss of function of P4HTM. The numbers represent amino acid position. TMD transmembrane domain.

In trio WGS, no candidate variants were identified in ‘diagnostic grade’ genes listed on the applied panels (Mitochondrial disorders v1.66 and Intellectual disability v2.111), through standard 100,000 Genome Project analysis pipelines. Through careful reanalysis, focusing on rare variants with predicted protein impact, we identified compound heterozygous variants in *P4HTM*. This gene had not been included in either of the applied panels as there was insufficient evidence of gene:disease association at the point of panel curation, which was prior to 2019 (Fig. 1). The maternally inherited frameshift, *P4HTM* NM_177938.2: c.[569_579del], p.(Gln190LeufsTer9) and paternally inherited stop gain, *P4HTM* NM_177938.2: c.[659G>A], p.(Trp220Ter) variants were confirmed by Sanger sequencing. Both variants were novel, absent from gnomAD v2.1.1 and predicted to lead to loss of function (due either to protein truncation with loss of both the EF-hand and the P4H alpha subunit domains or more likely due to nonsense mediated decay) in keeping with previously reported variants in patients with HIDEA syndrome. Details of genomic investigations undertaken and *P4HTM* variant interpretation using ACMG guidelines can be found in Supplementary Tables 1 and 2 respectively [7].

## Discussion

Biallelic loss of function *P4HTM* variants have been associated with HIDEA syndrome in 20 patients from 9 families previously. Of note, the P4HTM NM_177938.2 c.[1073G>A] variant has been reported in two Finnish families and appears to have an allele frequency 20 times higher in the Finnish population than non-Finnish Europeans in gnomAD [5, 8]. The P4HTM NM_177938.2 c.[949delG] variant has now been reported in two Syrian families [5, 9] suggesting a possible founder effect.

Table 1 [5, 8, 9] summarises the findings in the previously reported cases and phenotypic overlap with the patient we report. The predominance of males is presumed to be by chance, given the autosomal recessive nature of the condition. The clinical picture appears to be characterised by moderate to severe neonatal hypotonia, severe to profound intellectual disability, hypoventilation that was documented to be central in origin in 6 of the patients, recurrent pneumonias, strabismus, abnormal visual behaviour, and a high incidence of seizures (48%), but without significant structural brain or ocular abnormalities. Only nine of the eighteen patients that survived beyond infancy learned to walk, at between 1.5 and 5 years of age. Of the 16 for whom information was available, 8 never developed speech and 5 were reported to have poor or absent speech, while 3 were able to communicate using simple sentences.

Three patients, including the patient we report, died in infancy, following intercurrent respiratory infections in two. No cause was specified in the third. A further three patients died in childhood, two during febrile respiratory illnesses at 5 years and 8 years respectively, and one at 8 years of age from a respiratory arrest. The oldest reported patient died aged 61 years with acute pneumonia.

Evidence of hypoventilation was found in eight of the patients reported to date and in those who had formal sleep studies, it appeared to be central in origin. Hypoventilation in patients with neuromuscular disorders is often due to respiratory muscle weakness. Central hypoventilation is not usually associated with hypotonia. Most commonly it results from heterozygous polyalanine expansion mutations in *PHOX2B* [10], which may not be reliably detected in exome based testing. Specific testing for *PHOX2B* polyalanine expansion mutations in this patient was normal and no other variants in *PHOX2B* or other genes known to be associated with central hypoventilation were identified by WGS. Obstructive sleep apnoea requiring nocturnal BiPAP was also a common finding in the reported patients with HIDEA. Four of the previously reported patients were suspected to have a sleep behaviour disorder [5, 8].

In addition to refractive errors, strabismus and amblyopia (all common findings in children with neurological disorders and the general paediatric population), poor visual behaviour with roving or rotary eye movements was noted in 57% of the HIDEA patients. No consistent VEP or electroretinogram (ERG) abnormalities have been reported, but where findings were documented, fundal hypopigmentation was noted in 44% (7/16); temporal pallor in a further patient; and pale optic nerves or optic atrophy in 12% (2/17). Visual dysfunction has been reported frequently in patients with mitochondrial complex I deficiency [11].

Mitochondrial abnormalities have been documented in three of four previously reported patients with HIDEA who had muscle biopsies (Table 2). Muscle histology was normal in one (P1) who had low complex II + III activity (31% of control); but non-specifically abnormal (including ultrastructural mitochondrial abnormalities) in a second (P2) who had low activity of complexes I–III (41%) and IV (38%). COX-negative fibres were observed at post-mortem in one (P13). A fourth patient (P5) had myopathic histological features. Respiratory chain activities were not reported in these latter two patients [5].

**Table 2:** Muscle biopsy results of individuals reported with biallelic variants in *P4HTM*.

		Current patient	Rahikkala et al [5] and Kaasinen et al [8]			
			Family 1: P1	Family 2: P2	Family 3: P5	Family 5: P13
Age (Age deceased)		(7 months)	(5 years)	18 years	(8 years)	(61 years)
Age at muscle biopsy (where specified)		6 months	2.5 years	–	8.5 years	61 years
Muscle biopsy	Histology	Mild myopathic changes Significant abnormal variation in fibre size with type 2 predominance and coexpression of fast and slow myosin isoforms. Many fibres expressing neonatal myosin.	Normal histology	Electron microscopy showing increased mitochondria and some with enlarged and abnormal shapes	Increased variability in muscle fibre diameter, hypertrophied muscle fibres, scattered small atrophic fibres, basophilic muscle fibres	(Autopsy specimen) Unspecific type 2 muscle fibre atrophy and COX-negative fibres
	Respiratory chain enzyme testing	Reduced complex I to <30% of control mean Reduced complex IV to 50% of control mean (marginal) Normal complexes II and III	Reduced complex II + III activity to 31% of control	Reduced complexes I–III to 41% of control Reduced complex IV to 38% of control	NR	NR

*NR* not reported.

Muscle histology in the patient we report showed only mild myopathic fibre size variation. There were no ragged red or COX-negative fibres, hallmark features of mitochondrial disease that are rarely observed in childhood, even in genetically confirmed mitochondrial disorders [12]. However, respiratory chain enzyme assays revealed decreased complex I (NADH:ubiquinone oxidoreductase) activity at <30% of mean control, fitting published diagnostic criteria for a mitochondrial disorder [13]. In addition, complex IV activity was marginally decreased. The residual levels of mitochondrial respiratory chain complex I activity in the patient we report are similar to those seen in patients with primary mitochondrial disease caused by loss of function variants in subunits and assembly factors of complex I [14–16]. Together with the observation of mitochondrial dysfunction in previously reported patients with HIDEA syndrome, these findings lead us to postulate that P4HTM deficiency should be considered a mitochondrial disorder, although the precise molecular mechanisms leading to mitochondrial dysfunction remain to be unravelled. The distinction between primary and secondary mitochondrial disorders is the subject of much debate [17] but a widely accepted definition of a primary mitochondrial disorder is a genetic disorder leading to dysfunction of oxidative phosphorylation or other disturbances of mitochondrial structure, ultrastructure or function [18]. An increasing number of genetic disorders have been linked to secondary respiratory chain deficiency [17, 19].

The exact function of P4HTM is not known but it has been annotated as an endoplasmic reticulum (ER) transmembrane prolyl 4-hydroxylase (P4H) [20]. Prolyl 4-hydroxylation is a critical post-translational modification needed for the function of a diverse array of proteins [21]. Two groups of P4Hs are recognised: those that hydroxylate proline residues in collagen, and a second group of P4Hs that hydroxylate proline residues in hypoxia inducible factors (HIFs) [20]. HIFs are needed for the cellular response to hypoxia and 4-hydroxylation of critical proline residues targets HIFs for polyubiquitination and proteasomal degradation. Abnormal HIF-1α levels have recently been reported in some primary genetic mitochondrial disease [22, 23]. Targeting HIF P4Hs has been suggested as a strategy for neuroprotection [24, 25], but the presence of severe disease related to P4HTM deficiency suggests that caution should be exercised in exploring such a therapeutic approach. Other possible targets of P4HTM include the large subunit of RNA polymerase II and activating transcription factor 4 (ATF4) [20]. RNA polymerase II is needed for the transcription of all nuclear-encoded mRNAs, including the >1100 nuclear genes whose products are targeted to the mitochondrion [26]. Impaired function of RNA polymerase II as a result of abnormal prolyl 4-hydroxylation may lead to aberrant levels of hundreds of proteins needed for normal mitochondrial function. ATF4 has been implicated in the mitochondrial stress response in cellular and mouse models of mitochondrial disease [27, 28]. Thus, impaired prolyl 4-hydroxlation of HIF, RNA polymerase II or ATF4 could all plausibly account for the mitochondrial dysfunction observed in HIDEA patients and warrants further investigation. A final possibility is that, given its location in the ER membrane, P4HTM deficiency may cause mitochondrial dysfunction by perturbation of the mitochondria-associated membranes (MAMs), as is the case in MEGDEL syndrome, a form of Leigh syndrome caused by impaired lipid remodelling in the MAMs [29].

In summary, HIDEA syndrome is an important consideration in the investigation of profoundly hypotonic infants, particularly those with associated hypoventilation, poor visual behaviour or fundal hypopigmentation. Whilst management currently is principally supportive, a molecular diagnosis can inform counselling about prognosis and provide options for prenatal or preimplantation genetic diagnosis in subsequent pregnancies. Further research is warranted to elucidate the molecular basis, particularly of the mitochondrial dysfunction, and potential for specific disease-modifying therapies.

## Supplementary information

Supplementary material
