# Supplementary material for: Biallelic P4HTM variants associated with HIDEA syndrome and mitochondrial respiratory chain complex I deficiency
Source: Eur J Hum Genet. 2021 Jul 20;29(10):1536–41. doi: 10.1038/s41431-021-00932-8 (PMC8484625; doi:10.1038/s41431-021-00932-8)
Supplement: Supplementary file 1 — Supplementary material [file 41431_2021_932_MOESM1_ESM.docx]

**SUPPLEMENTAL MATERIAL**

**Figure S1. Sleep Study**

**↓**


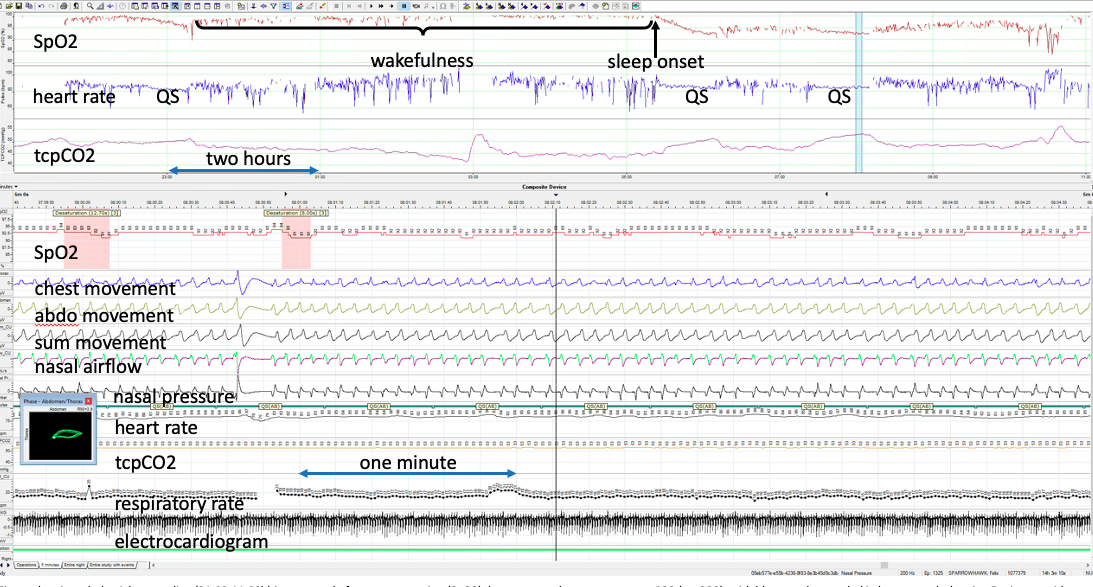


Baseline sleep study conducted at 2 months Upper Panel - Whole night recording (21:00-11:00h) illustrating oxygen saturation (SpO2), heart rate and transcutaneous pCO2 (tcpCO2). Arrowed region expanded in Lower Panel, showing five minutes with SpO2 (Nonin); chest, abdominal and sum respiratory movements; nasal flow and pressure; heart rate; tcpCO2 (Radiometer at 42°C); respiratory rate and electrocardiogram during Quiet Sleep (QS). The key findings are mild baseline hypoxaemia (SpO2 mean 93%), increased hypoxaemic episodes, bradypnoea and hypercapnia.

**Table S1. Genomic investigations**

| SINGLE GENE ANALYSIS AND ARRAY | | |
| --- | --- | --- |
| Disorder | **Gene** | **Result** |
| Congenital myotonic dystrophy | *DMPK* | No expansion |
| Prader-Willi syndrome | 15q11.2 methylation | Normal |
| Spinal muscular atrophy | *SMN1/2* MLPA | Normal |
| Alpers syndrome | *POLG (Common mutations)* | Normal |
| Congenital myasthenic syndrome | *DOK7* (exon 1-7)  *RAPSN* (exon 1-8, promotor) | Normal  Normal |
| Congenital central hypoventilation syndrome | *PHOX2B* (polyalanine expansion)  (full sequencing in panel below) | Normal |
| Array | *-* | Normal |

| MITOCHONDRIAL GENOME AND NUCLEAR MITOCHONDRIAL GENE ANALYSIS | |
| --- | --- |
| Subtest | **Result** |
| Large scale structural rearrangements | Normal |
| *MT-TL1* m.3243A>G | Normal |
| Mitochondrial DNA depletion analysis | Normal (68% mean) |
| 21 Panel mitochondrial DNA maintenance genes  *ABAT, AFG3L2, AGK, C10orf2, DGUOK, DNA2, FBXL4, MFN2, MGMW1, MPV17, OPA1, POLG, POLG2, RNASEH1, RRM2B, SLC25A4, SPG7, SUCLA2, TK2, TYMP* | No clear disease causing variants |
| 178 Panel of genes associated with mitochondrial disorders  *Nuclear mitochondrial disease*  *AARS2, ABCB7 ACAD9 AC02 ADCK3 ADCK4 AFG3L2 AGK AIFM1. ANO10 APOPT1 APTX ATPAF2 BCS1L BOLA3 BTD C10orf2 C12orf65 C19orf12 CHCHD10 CHKB CLPB CLPP COQ2 COQ4 COQ6 COQ9 COX10 COX14 COX15 COX20 COX6A1 COX6B1 COX7B CYC1 CYCS. DARS DARS2 DGUOK DHTKD1 DLAT DLD DNA2 DNAJC19 ECHS1 ELAC2 ETFDH ETHEL FARS2 FASTKD2 FBXL4 FH FOXRED1 G6PC GARS GATM GDAP1 GFM1 GLRX5 GLUD1 HCCS HIBCH HLCS HSPA9. HSPD1 KARS LARS2 LIAS LIPT1 LRPPRC MFN2 MGME1 MPV17 MRPS22 NDUFA1 NDUFA10 NDUFA11 NDUFA2 NDUFAF1 NDUFAF2 NDUFAF3 NDUFAF4 NDUFAF5 NDUFB11 NDUFS1 NDUFS2 NDUFS3 NDUFS4 NDUFS6 NDUFS7 NDUFS8 NDUFV1 NDUFV2 NFU1. NUBPL OPA1 PC PDHA1 PDHB PDHX PDP1 PDSS1 PDSS2 PET100 PMPCA PNPT1 POLG POLG2 PYCR1 QARS. RARS2 RMND1 RRM2B SACS SAMHD1 SC01 SC02 SDHA SDHAF1 SDHB SLC19A2 SLC19A3 SLC25A19 SLC25A22 SLC25A26 SLC25A3 SLC25A38 SLC25A4 SLC25A46 SPG7 SUCLA2 SUCLG1 SURF1. TAC01. TAZ. TIMM8A TK2 TMEM70. TPK1. TRMU TRNT1. TTC19 TUFM. TYMP UQCRB UQCRQ*  *Complex I deficiency*  *AARS2 CARS2 DARS2. EARS2 FARS2 HARS2 IARS2 LARS2 MARS2 NARS2 NDUFA1 NDUFA10 NDUFA11 NDUFA2 NDUFA3 NDUFA4 NDUFA5 NDUFA6 NDUFA7 NDUFA8 NDUFAF1 NDUFAF2 NDUFAF3 NDUFAF4 NDUFAF5 NDUFB1. NDUFB10 NDUFB11 NDUFB2 NDUFB3 NDUFB4 NDUFB5 NDUFB6 NDUFB7 NDUFB8 NDUFC1 NDUFC2 NDUFS1 NDUFS2 NDUFS3 NDUFS4 NDUFS5 NDUFS6 NDUFS7 NDUFS8 NDUFV1 NDUFV2 NDUFV3 PARS2 RARS2 SARS2 TARS2 VARS2 WARS2 YARS2* | No clear disease causing variants |

| WHOLE GENOME SEQUENCING (100,000 GENOMES PROJECT) |  |
| --- | --- |
| Subtest | **Result** |
| Mitochondrial disorders v1.66  *LACTB LARS LETM1 LIPT2 LYRM4 LYRM7 MPC1 MRPL12 MRPL3 MRPL40 MRPL44 MRPS16 MRPS2 MRPS23 MRPS7 MTFMT MTPAP NADK2 NDUFA12 NDUFA13 NDUFA3 NDUFA5 NDUFA6 NDUFA7 NDUFA8 NDUFA9 NDUFAB1 NDUFB1 NDUFB10 NDUFB2 NDUFB4 NDUFB5 NDUFB6 NDUFB7 NDUFB8 NDUFC1 NDUFC2 NDUFS5 NDUFV3 NFS1 NNT OGDH OXA1L PANK2 PDK1 PDK2 PDK3 PDK4 PDP2 PITRM1 POP1 PTCD1 PUS1 QRSL1 SDHAF2 SDHC SFXN4 SLC25A1 SLC25A12 SLC25A13 SLC25A40 SRRT STAT2 SUCLG2 TIMM44 TMEM126A TRAP1 TRIT1 TRMT10C TRMT5 TXN2 UQCC2 UQCC3 UQCRC1 UQCRC2 UQCRFS1 UQCRH WARS2 C1QBP* | No clear disease causing variants |
| Intellectual disability v2.111  *AAAS ACADS ACTL6A ADK AHI1 AMER1 AP3B1 AP3B2 ARID2 ARL13B ASL ASXL2 ATP6V0A2 B3GLCT BCS1L BMP4 BRPF1 BSCL2 CACNA1A CACNA1C CACNA1D CAMK2A CAMK2B CCDC88C CDH11 CDK13 CHD4 CHMP1A CIC CLCN4 CLTC COG5 COQ8A CRADD D2HGDH DAG1 DHX30 DIS3L2 DNAJC19 EBF3 ELP2 EML1 EMX2 FGF12 GABRG2 GFER GLIS3 GMPPB GPAA1 GRID2 HACE1 HECW2 HIVEP2 HNRNPH2 IARS IER3IP1 ITPR1 KCNJ6 KIAA0586 KIAA1109 KIDINS220 KIF1BP KMT2C KNL1 LARGE1 LINS1 LONP1 MBOAT7 MBTPS2 MDH2 NDST1 NEXMIF NFIA NTRK1 OPA3 PARN PDE4D PDHX PEX11B PGAP1 PIK3CA PLAA PLPBP PNPLA6 PRKD1 PRMT7 PRUNE1 PUF60 PUS1 PYCR1 PYCR2 QARS RAC1 RERE RLIM RRM2B RTTN SERAC1 SIN3A SLC33A1 SLC6A9 SMAD4 SMC3 SON SPART SPTBN2 ST3GAL5 STAG1 SZT2 TAF1 TBCD TBCK TECPR2 THOC2 THOC6 TMEM240 TMTC3 TRIP12 TRIT1 TRMT10A TTC37 UBA5 UBTF UNC80 VAMP1 WDR81 ZBTB18 ZSWIM6 AARS AASS ABCC9 ABCD1 ABCD4 ABHD5 ACAD9 ACADM ACO2 ACOX1 ACSL4 ACTB ACTG1 ACY1 ADAR ADGRG1 ADNP ADSL AFF2 AFF4 AFG3L2 AGA AGPS AHDC1 AIFM1 AIMP1 AKT3 ALDH18A1 ALDH3A2 ALDH4A1 ALDH5A1 ALDH7A1 ALG1 ALG11 ALG12 ALG13 ALG3 ALG6 ALG8 ALMS1 ALS2 AMPD2 AMT ANKH ANKRD11 AP1S2 AP4B1 AP4E1 AP4M1 AP4S1 APOPT1 APTX ARFGEF2 ARG1 ARHGEF6 ARHGEF9 ARID1A ARID1B ARL6 ARMC9 ARSA ARSB ARSL ARX ASAH1 ASPA ASPM ASXL1 ASXL3 ATAD3A ATIC ATM ATP13A2 ATP1A3 ATP6V1B2 ATP7A ATR ATRX AUH AUTS2 B3GALNT2 B4GALNT1 B4GALT7 BBS1 BBS10 BBS12 BBS2 BBS4 BBS5 BBS7 BBS9 BCAP31 BCKDHA BCKDHB BCL11A BCOR BLM BOLA3 BRAF BRAT1 BRWD3 BTD BUB1B C12orf57 C12orf65 C5orf42 CA2 CA8 CACNA1G CAMTA1 CASK CBL CBS CC2D1A CC2D2A CCBE1 CCDC22 CCDC8 CCND2 CDC6 CDH15 CDK5RAP2 CDKL5 CDON CDT1 CENPF CENPJ CEP135 CEP152 CEP290 CEP41 CEP57 CEP83 CHAMP1 CHD2 CHD7 CHD8 CISD2 CIT CKAP2L CLN3 CLN5 CLN6 CLN8 CLP1 CLPB CNKSR2 CNNM2 CNTNAP2 COASY COG1 COG4 COG7 COG8 COL4A1 COL4A2 COL4A3BP COLEC11 COQ4 COX10 COX15 COX6B1 CPS1 CRB2 CREBBP CSNK2A1 CSPP1 CSTB CTC1 CTCF CTDP1 CTNNB1 CTSA CTSD CUL4B CYB5R3 CYC1 CYP2U1 DARS DARS2 DBT DCAF17 DCHS1 DCX DDC DDHD2 DDOST DDX11 DDX3X DEAF1 DEPDC5 DHCR24 DHCR7 DHFR DHTKD1 DIAPH1 DKC1 DLD DLG3 DMD DNAJC12 DNM1 DNMT3A DNMT3B DOCK7 DOCK8 DOLK DPAGT1 DPM1 DPP6 DPYD DYM DYNC1H1 DYRK1A EBP EDNRB EED EEF1A2 EFTUD2 EHMT1 EIF2AK3 EIF2S3 ELAC2 ELOVL4 EP300 EPG5 ERCC1 ERCC2 ERCC3 ERCC5 ERCC6 ERCC6L2 ERCC8 ESCO2 ETFA ETFB ETFDH ETHE1 EXOSC3 EXTL3 EZH2 FAM111A FAM126A FAM20C FAR1 FAT4 FBXL4 FGD1 FH FIG4 FKRP FKTN FLNA FLVCR1 FLVCR2 FMN2 FMR1 FOLR1 FOXG1 FOXP1 FOXP2 FOXRED1 FTCD FTSJ1 FUCA1 GABRA1 GABRB3 GALC GALE GALT GAMT GATAD2B GATM GCDH GCH1 GDI1 GFAP GFM1 GJC2 GK GLB1 GLDC GLI2 GLI3 GLUL GLYCTK GM2A GMPPA GNAO1 GNAS GNB1 GNPAT GNPTAB GNPTG GNS GPC3 GPSM2 GRIA3 GRIK2 GRIN1 GRIN2A GRIN2B GRM1 GSPT2 GTF2H5 GTPBP3 GUSB HADH HADHA HCCS HCFC1 HCN1 HDAC4 HDAC8 HERC1 HESX1 HEXA HEXB HGSNAT HIBCH HIST1H1E HLCS HMGCL HNRNPU HOXA1 HPD HPRT1 HRAS HSD17B10 HSD17B4 HSPD1 HTRA2 HUWE1 HYLS1 IDH2 IDS IDUA IFIH1 IFT172 IGF1 IKBKG IL1RAPL1 INPP5E INPP5K IQSEC2 IRX5 ISPD IVD JAM3 KANSL1 KAT6A KAT6B KCNA2 KCNB1 KCNC1 KCNC3 KCNH1 KCNJ10 KCNJ11 KCNK9 KCNQ2 KCNT1 KCTD7 KDM5B KDM5C KDM6A KIF11 KIF1A KIF5C KIF7 KMT2A KMT2D KPTN KRAS L1CAM L2HGDH LAMA1 LAMA2 LAMB1 LAMC3 LAMP2 LARP7 LGI4 LIG4 LRP2 LRPPRC MAB21L2 MAF MAGEL2 MAN1B1 MAN2B1 MANBA MAOA MAP2K1 MAP2K2 MASP1 MAT1A MBD5 MCCC1 MCCC2 MCOLN1 MCPH1 MECP2 MED12 MED13L MED17 MEF2C MFF MFSD8 MGAT2 MGP MICU1 MID1 MKKS MKS1 MLC1 MLYCD MMAA MMAB MMACHC MMADHC MOCS1 MOCS2 MOGS MPDU1 MPI MPLKIP MRPS22 MSMO1 MTHFR MTOR MTR MTRR MUT MVK MYCN MYO5A MYT1L NAA10 NACC1 NAGA NAGLU NALCN NANS NDE1 NDP NDUFA1 NDUFS1 NDUFS4 NDUFS7 NDUFS8 NDUFV1 NEU1 NF1 NFIX NFU1 NGLY1 NHS NIPBL NKX2-1 NLGN3 NONO NPC1 NPC2 NPHP1 NR2F1 NRAS NRXN1 NRXN2 NSD1 NSDHL NSUN2 NT5C2 NUBPL OCLN OCRL OFD1 OGT OPHN1 ORC1 ORC4 ORC6 OTC OTUD6B OTX2 PACS1 PAFAH1B1 PAH PAK3 PAX6 PAX8 PC PCCA PCCB PCDH19 PCGF2 PCNT PDHA1 PDSS1 PDSS2 PEPD PEX1 PEX10 PEX12 PEX13 PEX14 PEX16 PEX19 PEX2 PEX26 PEX3 PEX5 PEX6 PEX7 PGAP2 PGAP3 PGK1 PHF6 PHF8 PHGDH PIGA PIGL PIGN PIGO PIGT PIGV PIK3R2 PLA2G6 PLCB1 PLK4 PLP1 PMM2 PNKP POGZ POLG POLR3A POLR3B POMGNT1 POMGNT2 POMT1 POMT2 PORCN PPM1D PPP1CB PPP2R1A PPP2R5D PPT1 PQBP1 PRODH PRPS1 PRRT2 PRSS12 PSAP PSMD12 PSPH PTCH1 PTCHD1 PTDSS1 PTEN PTF1A PTPN11 PTS PURA QDPR RAB11B RAB18 RAB23 RAB39B RAB3GAP1 RAB3GAP2 RAD21 RAF1 RAI1 RANBP2 RARB RARS2 RBM10 RELN RFT1 RIT1 RMND1 RNASEH2A RNASEH2B RNASEH2C RNASET2 ROGDI ROR2 RPGRIP1L RPL10 RPS6KA3 RTEL1 SAMD9 SAMHD1 SATB2 SC5D SCN1A SCN2A SCN8A SCO1 SCO2 SDHA SDHAF1 SETBP1 SETD5 SGPL1 SGSH SHANK1 SHANK2 SHANK3 SHH SHOC2 SIK1 SIL1 SIX3 SKI SLC12A5 SLC12A6 SLC13A5 SLC16A2 SLC17A5 SLC19A3 SLC22A5 SLC25A1 SLC25A15 SLC25A20 SLC25A22 SLC2A1 SLC35A2 SLC35C1 SLC39A14 SLC39A8 SLC46A1 SLC4A4 SLC6A1 SLC6A17 SLC6A19 SLC6A3 SLC6A8 SLC9A6 SLX4 SMARCA2 SMARCA4 SMARCB1 SMARCE1 SMC1A SMOC1 SMPD1 SMS SNAP29 SNRPB SNX14 SOS1 SOX10 SOX11 SOX2 SOX3 SOX5 SOX9 SPATA5 SPG11 SPR SPRED1 SPTAN1 SRCAP SRD5A3 ST3GAL3 STAMBP STIL STRA6 STX1B STXBP1 SUCLG1 SUMF1 SUOX SURF1 SYN1 SYNGAP1 SYNJ1 SYP TANGO2 TAT TAZ TBC1D24 TBCE TBL1XR1 TBR1 TCF4 TCN2 TCTN2 TGIF1 TH THAP1 THRA TIMM8A TLK2 TMCO1 TMEM165 TMEM216 TMEM237 TMEM5 TMEM67 TMEM70 TOE1 TPP1 TRAPPC9 TREX1 TRIM32 TRIM37 TRIO TSC1 TSC2 TSEN2 TSEN34 TSEN54 TSFM TSHB TSPAN7 TTC19 TTC8 TTI2 TUBA1A TUBA8 TUBB TUBB2A TUBB2B TUBB3 TUBB4A TUBGCP6 TUSC3 TWIST1 UBE2A UBE3A UBE3B UBR1 UMPS UPF3B UROC1 USP9X VLDLR VPS13B VRK1 WAC WDPCP WDR45 WDR45B WDR62 WDR73 WWOX XRCC4 YWHAG YY1 ZBTB20 ZC4H2 ZDHHC9 ZEB2 ZFYVE26 ZIC2 ZMYND11 ZNF711 ABCB11 ABCC6 ACADVL ACAN ACAT1 ACP5 ACTA2 ACVR1 ADA ADCY5 ADGRG6 AGL AGXT AIPL1 AIRE AK2 AKR1D1 AKT1 ALAD ALDOA ALDOB ALPL ALX3 ALX4 ANO5 ANTXR1 ARL14EP ARMC4 ASH1L ASS1 ATP1A2 ATP6AP2 ATP6V1B1 ATP8B1 B3GALT6 BFSP2 BGN BHLHA9 BICD2 BMPER BMPR1B BSND C19orf12 C2CD3 C2orf71 C4orf26 C8orf37 CARS2 CASR CCDC103 CCDC114 CCDC40 CCDC65 CCNO CCT5 CD96 CDH23 CDH3 CDK16 CDKN1C CEP104 CEP63 CHL1 CHM CHRDL1 CHRNG CHST14 CHST3 CHSY1 CHUK CIB2 CLCN2 CLCN7 CLDN19 CNOT3 COL10A1 COL11A1 COL18A1 COL1A1 COL2A1 COL4A3 COL4A4 COL6A1 COL9A1 COL9A2 COL9A3 COMP COQ9 COX7B CRB1 CRBN CRX CRYAA CRYBA1 CRYBB1 CRYBB2 CRYBB3 CRYGD CTNS CTSF CTSK CYP1B1 CYP27A1 CYP7B1 DCC DDB2 DDX53 DENND5A DIP2B DLAT DLG1 DLG2 DLG4 DLL3 DLL4 DMP1 DNAAF3 DNAAF4 DOCK6 DPM3 DSPP DSTYK DVL1 DYNC2H1 ECEL1 EDA EDNRA EEF1B2 EIF4A3 ELN ENPP1 EOGT EPB41L1 ERCC4 ERF ERLIN2 ERMARD EVC EVC2 EXT2 EYA1 FA2H FAAH2 FAM120C FAM161A FAM20A FANCA FANCC FANCD2 FANCE FANCF FANCG FANCI FBN1 FBP1 FBXW4 FGF10 FGF14 FGF3 FGFR2 FHL1 FKBP14 FLNB FLT4 FOXC1 FOXC2 FOXE1 FOXE3 FOXF1 FOXN1 FRAS1 FREM2 FRMPD4 FTL FTO FXN FYCO1 FZD6 GAA GABBR2 GALK1 GAS8 GATA2 GATA4 GATA6 GBA GBA2 GDF5 GDF6 GHR GJA1 GJA3 GJA8 GJB1 GJB2 GJB3 GLE1 GLMN GNAI1 GNAI3 GPR179 GRHL3 GRIA1 GRM6 GSS GUCY2C HAX1 HINT1 HIST1H4C HMGCS2 HNF1B HNF4A HNRNPK HOXA13 HOXC13 HOXD13 HPGD HPS1 HPSE2 HR HSD3B7 HSF4 HSPG2 HTT HYAL1 HYDIN IFITM5 IFT122 IFT43 IFT80 IGF1R IGF2 IHH IL11RA IL1RAPL2 IMPAD1 INPPL1 IRF6 ITGA7 JAG1 JAGN1 JAK3 KARS KBTBD13 KCND3 KCNQ1 KCTD1 KIF22 KIF2A KIRREL3 KIT KLF1 KLHL15 KLHL40 KMT2B LDB3 LEMD3 LFNG LHX3 LHX4 LMBRD1 LMNA LMX1B LRP4 LRP5 LRRC6 LTBP2 LTBP3 MAP3K1 MAPK10 MAPRE2 MATN3 MC2R MED23 MED25 MESP2 MFRP MFSD2A MIR17HG MMP13 MNX1 MSX1 MSX2 MTO1 MYH6 MYH8 MYH9 MYO5B NAA15 NAGS NBAS NBN NDUFAF2 NEK1 NKX3-2 NMNAT1 NODAL NOG NOTCH2 NPHP3 NPHP4 NPHS1 NPR2 NR2F2 NR5A1 NT5C3A NUP107 NUP62 NYX OTOGL OTULIN PAPSS2 PAX3 PAX9 PDE6G PET100 PGM1 PGM3 PHF21A PIK3R1 PITX2 PITX3 PKD1L1 PKHD1 PLOD2 PMS2 PNPO PNPT1 POC1B POLD1 POLR1C POLR1D POU1F1 PPA2 PRDM12 PROP1 PRSS56 PSMB8 PTH1R PTHLH QRICH1 RASA1 RAX RBPJ RETREG1 RMRP RNF135 RNU4ATAC ROBO3 RPE65 RPGRIP1 RPS19 RPS23 RSPH1 RSPH3 RSPO4 RTN4IP1 RUNX2 SACS SALL1 SALL4 SBDS SCARF2 SCN11A SCN4A SDCCAG8 SEC23B SET SETD1A SH3PXD2B SHROOM4 SIX1 SIX5 SKIV2L SLC25A26 SLC25A38 SLC26A2 SLC27A4 SLC2A2 SLC35D1 SLC39A13 SLC4A1 SLC4A11 SLC5A5 SMAD3 SMARCD2 SMCHD1 SNORD118 SOX17 SPAG1 SPEG SRGAP3 SRPX2 SRY STAR STAT1 STRADA STS STT3A TAB2 TAF13 TBX1 TBX15 TBX20 TBX22 TBX3 TBX4 TBX5 TBXAS1 TCF12 TCF20 TCTN3 TEK TERT TGDS TGFB1 TGFB2 TGFB3 THRB TMEM126B TMEM231 TMPRSS6 TP63 TRAPPC11 TRAPPC2 TRIP11 TRMT1 TRPM1 TRPS1 TRPV4 TSHR TUBGCP4 TWIST2 TXNL4A TYR TYRP1 UGT1A1 UPB1 UROS USB1 USP27X UVSSA VIPAS39 VPS33B VSX2 WASHC5 WDR19 WDR34 WDR35 WNT1 WNT10B WNT3 WNT7A WRAP53 XPA XPC XYLT1 ZC3H14 ZFP57 ZIC1 ZIC3 ZMPSTE24 ZNF148 ZNF335 A2ML1 ABAT ABCB7 ABHD12 ACBD6 ACE2 ACIN1 ACOT9 ACSF3 ACTL6B ADGRG4 ADGRV1 ADRA2B AGK AGO1 AGPAT2 AGTR2 AHCY AK1 AKAP17A AKAP4 AKAP6 AKR1C2 ALDH1A3 ALG2 ALG9 ALX1 ANK3 ANO10 ANO3 AP5Z1 AR ARHGAP31 ARHGAP36 ARHGAP6 ARHGEF2 ARHGEF4 ARIH1 ARSF ASB12 ASCC3 ASCL1 ASMT ASMTL ATCAY ATL1 ATN1 ATP2A2 ATP2B3 ATP7B ATP8A2 ATXN1 ATXN10 ATXN2 ATXN3 ATXN3L ATXN7 AVPR2 AWAT2 B4GALT1 B9D1 BCORL1 BDP1 BEAN1 BIN1 BMP15 BPIFB6 BRCA1 BRCA2 BRIP1 BTK C9orf72 CACNA1F CACNA1H CACNA1S CACNB4 CACNG2 CAMK2G CAP1 CAPN10 CAPRIN1 CASP2 CCDC115 CCDC39 CCDC78 CCNA2 CCNB3 CD99 CDC45 CDK19 CDK8 CFAP47 CFP CHRNA2 CHRNA4 CHRNB2 CLCN5 CLCNKA CLCNKB CLIC2 CMC4 CNKSR1 COA5 COL11A2 COL4A6 COL6A3 COQ2 COQ5 COX14 CP CPA6 CPXCR1 CRLF2 CRYBA4 CRYGC CSF1R CSF2RA CSNK1G1 CSTF2 CTPS2 CTTNBP2 CUL7 CUX2 CXorf58 DACT1 DCHS2 DCTN1 DDHD1 DDR2 DECR1 DGKH DHODH DHRSX DIAPH2 DMPK DMXL2 DNA2 DNM2 DNMT1 DOCK11 DPF1 DPF2 DPF3 DRD2 DST DVL3 EFHC1 EFNB1 EGR2 EIF4G1 ELK1 ELOVL5 ENOX2 ENTPD1 EOMES EPM2A EPPK1 ESX1 EXT1 FAH FAM111B FAM47B FAM58A FANCB FASN FBN2 FBXO25 FBXO7 FBXO8 FGD4 FGFR1 FGFR3 FKBP6 FKBPL FLAD1 FOXP3 FREM1 FRMD7 FRY GAB3 GABRQ GAD1 GALNS GCSH GDAP1 GLRA1 GLRA2 GLUD1 GNAL GON4L GORAB GOSR2 GPHN GPRASP1 GRB14 GRIA2 GRN GTPBP8 HAUS7 HDAC6 HIST1H4B HIST3H3 HK1 HMGB3 HS6ST2 IARS2 IFNAR2 IFT140 IGBP1 IGHMBP2 IGSF1 IL3RA INF2 INPP4A INTS6L IRAK1 ITCH ITGA3 ITGA4 ITGB6 ITIH6 KANK1 KCNA1 KCND1 KCNE1 KCNH5 KCNK12 KCNMA1 KCNQ3 KCNQ5 KDM1A KDM5A KDM6B KIF1C KIF26B KIF4A KIF5A KLF8 KLHL21 KLHL34 KLHL4 KRIT1 LAS1L LBR LGI1 LHFPL3 LIMK1 LITAF LOXHD1 LRAT LRP1 LRRK1 LRRK2 LYST MAFB MAGEA11 MAGEB1 MAGEB10 MAGEB2 MAGEC1 MAGEC3 MAGED1 MAGEE2 MAGI2 MAGIX MAGT1 MAOB MAP3K15 MAP7D3 MAPT MARS2 MBNL3 MCEE MECR MEGF10 MEGF8 MGAT5B MIB1 MITF MLH1 MMP21 MORC4 MPDZ MPV17 MPZ MRE11 MSL3 MT-ATP6 MT-ND1 MT-ND4 MT-TK MT-TP MTF1 MTM1 MTMR1 MTMR14 MTMR2 MTMR8 MTPAP MTTP MXRA5 MYBPC1 MYH10 MYH3 MYO1D MYO1G MYO7A NADK2 NDN NDRG1 NDUFA10 NDUFA11 NDUFA12 NDUFA9 NDUFAF3 NDUFS2 NDUFS3 NEB NECAB2 NECTIN1 NEDD4L NEFL NHEJ1 NHLRC1 NHP2 NIPA1 NKAP NKX2-5 NLGN4X NLRP3 NOP56 NPHS2 NR1I3 NRK NRXN3 NTM NXF4 NXF5 OBSL1 ODF2L OR5M1 OXCT1 P2RY4 P2RY8 P3H1 P4HB PABPC5 PALB2 PANK2 PARK7 PARP1 PASD1 PAX2 PBRM1 PCBD1 PCDH10 PCDH12 PCYT1A PDCD10 PDGFB PDGFRB PDYN PECR PGRMC1 PHACTR1 PHC1 PHF10 PHIP PHKA1 PHOX2B PIEZO2 PIGH PIGQ PIK3C3 PIN4 PINK1 PJA1 PLCE1 PLCXD1 PLEC PLOD1 PLOD3 PLXNB3 PMP22 PNKD PNP POC1A POLA1 PPOX PPP2R2B PRDX4 PREPL PRICKLE1 PRICKLE2 PRICKLE3 PRKAR1A PRKCG PRKN PRKRA PRMT9 PROX2 PRRG1 PRRG3 PRX PSAT1 PSEN1 PSMA7 PSMD10 PTPN21 PUDP PYGL QKI RAB27A RAB40AL RABL6 RAD50 RAD51 RAD51C RALGDS RAPGEF1 RAPSN RBM28 RBM8A RECQL4 REEP1 REEP2 RENBP RET RFX6 RGN RGS7 RHEB RIPK4 RNF113A RNF168 RNF216 RPGR RRAS RTL9 RTN2 RUBCN RYR1 RYR3 SBF2 SCAPER SCARB2 SCN1B SCN9A SCRIB SETDB2 SETX SF3B4 SGCE SH3TC2 SHOX SHROOM2 SIGMAR1 SLC20A2 SLC25A19 SLC25A53 SLC25A6 SLC26A9 SLC2A10 SLC30A9 SLC31A1 SLC35A1 SLC45A1 SLC52A3 SLC6A5 SLC9A9 SMARCA1 SMARCAL1 SMARCC1 SMARCC2 SMARCD1 SMARCD3 SMO SNCA SNIP1 SNTG1 SNX3 SOBP SPAST SPG21 SPG7 SPRY3 SPTLC2 SREBF2 STAB2 STARD8 STT3B STUB1 SVBP SYNCRIP SYNE1 SYT1 SYT14 SYTL4 SYTL5 TACO1 TAF2 TAF7L TANC2 TARDBP TBC1D8B TBP TCEAL3 TCOF1 TCP10L2 TCTN1 TECR TENM1 TEPSIN TFAP2A TFAP2B TFB2M TFG TGFBR1 TGFBR2 TGM6 THUMPD1 TINF2 TK2 TKTL1 TLR8 TM4SF20 TMEM132E TMEM135 TMLHE TNKS2 TNPO2 TOR1A TREX2 TRIP13 TSC22D3 TTBK2 TTC7A TTN TTPA TUBAL3 TUFM UBR7 UQCRB UQCRQ UTP14A VAMP7 VDR VIP VPS35 WDR11 WDR13 WDR4 WDR60 WNK3 WNT4 WNT5A WT1 WWC3 XIAP XK XKRX XPNPEP3 YAP1 ZBTB16 ZBTB40 ZCCHC12 ZCCHC8 ZDHHC15 ZFHX4 ZFX ZMYM3 ZMYM6 ZMYND12 ZNF41 ZNF425 ZNF526 ZNF592 ZNF599 ZNF674 ZNF713 ZNF81 AP1S1 ARCN1 ARV1 BCKDK BPTF BRF1 C12orf4 CCDC88A CDC42 CHKB CNTN3 CSNK2B CWC27 DOCK3 DPM2 FIBP GEMIN4 GRIA4 GTF3C3 HEPACAM HERC2 INTS1 INTS8 ISCA2 ITPA KCTD3 KIF14 KLHL7 KMT5B MEIS2 MTFMT NDUFAF5 PBX1 PCLO PIGC PIGW PIGY PPP1R15B PPP3CA PTPN23 RBBP8 RNF125 SCN3A SETD1B SETD2 SLC25A12 SPECC1L SSR4 TAF6 TBC1D23 TNIK TRAPPC6B UNC13A VPS53 WDFY3 WDR26 ZBTB24 FMR1_CGG ATXN10_ATTCT ATXN1_CAG ATXN2_CAG ATXN3_CAG ATXN7_CAG C9orf72_GGGGCC CSTB_CCCCGCCCCGCG DMPK_CTG FXN_GAA PPP2R2B_CAG* | No clear disease causing variants |
|  |  |
| Genes with variants identified through the 100,000 Genomes Project standard analysis  *ACSL4* c.1448A>G X-linked recessive mental retardation 63, MIM 300157  (maternal) Discounted as phenotype in patient not in keeping | |
| Genes with variants identified following manual reanalysis  *P4HTM* c.659G>A (paternal) Hypotonia, hypoventilation, impaired intellectual,  *P4HTM* c.569_579del (maternal) development, dysautonomia, epilepsy, and eye  abnormalities (HIDEA), MIM 618493 | |
|  |  |

**Table S2. Genomic interpretation *P4HTM***

**NM_177938.2: *P4HTM*** **c.659G>A, p.(Trp220Ter) (paternal)**

P4HTM 3:49002531 G > A

| Stop Gain at position 220 in exon 4 Class 5 Pathogenic |
| --- |
| PVS1  Null variant (nonsense) in a gene (*P4HTM*) where loss of function (LOF) variants are a known mechanism of disease |
| PM2  Absent from gnomAD genomes with good (>20 reads) genomes coverage (33.7 reads). |
| PM3  *in trans* with pathogenic variant |
| PP4_Moderate  Patient’s phenotype is highly specific for HIDEA, a disease with a single known genetic aetiology (biallelic LOF variants in *P4HTM*) |

**Variants classified in accordance with ACMG criteria, as outlined by Richards et al.** Genetics in Medicine 2015 17(5):405–24. https://doi.org/10.1038/gim.2015.30

**NM_177938.2: *P4HTM***: **c.569_579del, p.(Gln190LeufsTer9) (maternal)**

P4HTM 3:49001568 CCAGCTGGACCT > C

| Frameshift at position 190 in exon 3 Class 5 Pathogenic |
| --- |
| PVS1  Null variant (frameshift) in a gene (*P4HTM*) where loss of function (LOF) variants are a known mechanism of disease |
| PM2  Variant not found in gnomAD exomes with good (>20 reads) exome coverage (62.1 reads). |
| PM3  *in trans* with pathogenic variant |
| PP4_Moderate  Patient’s phenotype is highly specific for HIDEA, a disease with a single known genetic aetiology (biallelic LOF variants in *P4HTM*) |

**Variants classified in accordance with ACMG criteria, as outlined by Richards et al**. Genetics in Medicine 2015 17(5):405–24. https://doi.org/10.1038/gim.2015.30
